# Mutational probing of protein aggregates to design aggregation‐resistant proteins

**DOI:** 10.1002/2211-5463.12003

**Published:** 2016-01-04

**Authors:** Mohamad Zahid Kamal, Virender Kumar, Kundarapu Satyamurthi, Kushal Kumar Das, Nalam Madhusudhana Rao

**Affiliations:** ^1^ Centre for Cellular and Molecular BiologyCouncil of Scientific and Industrial ResearchHyderabadIndia

**Keywords:** aggregation resistance, intermediates, local unfolding, mutations, protein stability

## Abstract

Characterization of amorphous protein aggregates may offer insights into the process of aggregation. Eleven single amino acid mutants of lipase (LipA of *Bacillus subtilis*) were subjected to temperature‐induced aggregation, and the resultant aggregates were characterized for recovery of activity in the presence of guanidinium chloride (GdmCl). Based on activity recovery profiles of the aggregates, the mutants could be broadly assigned into four groups. By including at least one mutation from each group, a mutant was generated that showed an increase of ~ 10 °C in melting temperature (*T*_m_) compared to the wild‐type and did not aggregate even at 75 °C. This method explores characterization of amorphous protein aggregates in the presence of GdmCl and helps in identifying mutations involved in protein aggregation.

**Abbreviations**
GdmCl: guanidinium chloride
PNPB: p‐nitrophenyl butyrate
TfT: thioflavin T
*T*_m_: melting temperature
ΔG_unfold_: free energy of unfolding

Aggregation of proteins during expression, purification, formulation and storage is one of the major factors that limit their application. Protein aggregation is also associated with many pathological conditions in humans 1. Protein aggregation leading to amyloid aggregates has been extensively investigated 1, 2, 3. Mechanistically, aggregation in proteins is considered to be initiated by partially unfolded proteins, often termed as non‐native aggregation 4, 5, 6. Free energy of unfolding in proteins is small and at any given time a fraction of protein, albeit small, exists as partially unfolded state leading to aggregation. This equilibrium between folded and unfolded state in proteins is sensitive to changes in temperature, pH, ionic strength, protein concentration, surfaces etc. 7. As protein aggregation is a wasteful process leading to loss of protein in protein suspensions, development of methods that can prevent aggregation have significant economic benefit 8, 9. Optimizing temperature, excipients, pH etc. was found to be successful in preventing aggregation; however, success of any method in preventing protein aggregation can largely depend on nature of protein and the aggregation mechanism 10.

Protein aggregation primarily involves intermolecular interactions and initiates on the protein surface. Loops, or unordered regions of proteins, are located on the surface and are the most flexible structures in a protein. Loops display pronounced dynamics compared to the rest of the protein and often participate in functional protein‐protein interactions. The loop dynamics depends on the loop length, amino acid composition and its interaction with the rest of the structure. Upon exposure to heat or in nonphysiological conditions, the dynamics of the protein loops increases further leading to unfolding. One of the consequences of unfolding is exposure of the hydrophobic portion of the protein and interaction of these hydrophobic portions leads to protein aggregation. Detailed studies of several groups, including those of Dobson, Chiti and Ventura, on single amino acid variants of proteins have provided useful predictive rules linking proteins sequence(s) with protein aggregation kinetics 1, 2, 3, 9.

One approach to designing an aggregation‐resistant protein is by increasing the protein stability. Some regions of a protein are more susceptible to unfolding than other regions and thus act as sites for aggregation. By altering the dynamics in a protein by a mutation, for example, by replacing an amino acid with proline, one could alter the formation of an unfolding intermediate, which may result in a protein aggregate dissimilar to the aggregate formed by the wild‐type protein. 2. In this study, we have investigated the nature of protein aggregates generated by heat denaturation of a set of lipase mutants, which differ from each other by one amino acid, by monitoring the recovery of enzyme activity of the aggregate in the presence of a denaturant. The objective is to identify mutations that have stronger bearing on aggregation and use these mutations to design a protein that is more aggregation‐resistant than the wild‐type.

*Bacillus subtilis* lipase (Lip A) is used as a model protein in this study. It is a 181 amino acid long protein with an apparent melting temperature (*T*_m app._) of ~ 56 °C that undergoes complete irreversible aggregation upon heating 11, 12. The aggregates formed by heat denaturation of lipase are inactive and the activity could be regained in the presence of denaturants such as urea or guanidinium chloride (GdmCl) 13. The activity recovery profiles of the aggregates, generated from different mutants, show different patterns. Based on these patterns, we identified six mutations in lipase and combined them to design an aggregation‐resistant lipase. The study uses the activity profiles as a basis for identifying mutants and does not require the physical description of the aggregate.

## Materials and methods

All lipase variants were purified using the method described earlier 11, 12 and quantified by the modified Lowry method 14.

### Estimation of protein aggregation

Lipase variants (0.05 mg·mL^−1^ in 50 mm sodium phosphate buffer, pH 7.2) were heated at desired temperature for 20 min using a thermal block followed by cooling at 4 °C for 30 min and centrifugation at 22 000 ***g*** to remove any insoluble protein. Aggregation resistance or reversibility in the presence of GdmCl was estimated by measuring residual activity. Enzyme activity was determined at 25 °C using p‐nitrophenyl butyrate (PNPB) as substrate on a Perkin Elmer Lambda‐35 spectrophotometer attached with PTP‐1 Peltier temperature programmer as described earlier 11.

Light scattering experiments were performed on a Fluorolog 3–22 fluorimeter to capture the aggregation process. Lipase variants (0.05 mg·mL^−1^) were subjected to high temperature, and scattering was monitored at 360 nm as a function of time.

### Activity recovery of protein aggregates

Aggregates of lipase were formed by heating 75 μL of (0.05 mg·mL^−1^) lipase variants in 50 mm phosphate buffer pH 7.2 in a thermal block at 65 °C for 20 min, followed by cooling to 4 °C. The aggregates formed were then subjected to overnight incubation at varying concentrations (0–4.2 m) of guanidinium hydrochloride (GdmCl) at 4 °C for solubilization. In the next step, samples were diluted 5 fold and incubated at 4 °C for ~ 3 h. Enzyme activity was estimated as described earlier.

### Thermal unfolding

Thermal unfolding of lipase variants (0.05 mg·mL^−1^ in 50 mm sodium phosphate buffer, pH 7.2) was performed on a circular dichroism spectrophotometer (JASCO J‐815 spectropolarimeter) fitted with Jasco Peltier‐type temperature controller (CDF‐426S/15) in a 1 cm path length cuvette. Thermal unfolding profiles were obtained by heating the protein at a constant rate of 1 °C per minute while measuring the change in ellipticity at 222 nm.

### Thioflavin T binding assay

To protein aggregates of lipase and insulin (positive control) generated from a protein sample at 50 μg per ml, Thioflavin T (TfT, Final concentration 10 μm) was added in 100 mm Tris.Cl buffer at pH 8 15. Thioflavin T fluorescence emission was measured (Ex. 450 nm and EM. 480 nm) using a HITACHI F‐7000 fluorescence spectrometer. Insulin amyloids were generated by dissolving 2 mm of insulin in water (pH adjusted to 2 with HCl) and heated to 60 °C for several hours and cooled to room temperature.

## Results

### Identification of mutations that affect aggregation

Lipase (*Bacillus subtilis*) aggregates upon heating at temperatures above 50 °C and its thermal transition (thermal denaturation curve) is highly cooperative 11, 12, 13. However, the aggregated protein does not renature upon cooling. This is a normal behaviour shown by many proteins undergoing aggregation 16, 17, 18.

In earlier work, site‐saturation mutagenesis was performed at all the loop positions (86 positions) of lipase to identify mutations that can stabilize the protein 19. In the process, 86 of 181 amino acids residues in a lipase were converted to all other 19 residues by site‐saturation mutagenesis. Seventeen single mutants at 15 positions were identified that could increase the melting temperature (*T*_m_) by 1–6 °C while improving thermodynamic stability of native structure by 0.04–1.16 kcal·mol^−1^ (Table 1, 19).

**Table 1:** Scores of wild‐type and mutant lipase using four softwares on aggregation

Mutant	tango	gap	pasta	aggrescan	Half‐life (min)^a^	ΔG_H20_, kcal·mol^−1^ a
WT	831.904	0.862	20	−0.2	3.1	10.4
A15S	831.913	0.863	20	−0.4	19.5	10.6
F17T	831.169	0.866	20	−1.3	26.3	10.9
R33P	630.12	0.864	20	0.3	5.9	10.4
T47S	831.793	0.864	20	−0.3	25.3	11.4
T109V	831.91	0.854	20	0.7	36	10.7
G111S	831.905	0.86	20	−0.1	6.2	10.5
L114P	831.904	0.865	20	−1.2	8.7	10.6
A132D	811.313	0.854	20	−1.2	6.0	10.0
M134E	618.073	0.856	16	−1.5	196.0	11.0
M137P	557.289	0.861	15	−0.9	2617	11.6
N174T	831.902	0.872	20	0.6	9.4	10.8
GROUP 1	617.489	0.843	14	−3.2		
GROUP2	831.185	0.866	20	−1.4		
GROUP3	831.905	0.865	20	−1		
GROUP4	630.126	0.856	20	1.2		
RM3.1	556.556	0.864	14	−1.8		
RM3.2	617.489	0.843	14	−3.2		
RM6	556.576	0.86	14	−2		

Half‐life was determined by incubating the pure proteins at 55 °C and monitoring their activity till it reaches 20% of the initial activity. The slopes of ln(activity) vs. time are provided.

tango (http://tango.crg.es/) statistical mechanics algorithm based on simple physicochemical principles of secondary structure formation. gap (http://www.iitm.ac.in/bioinfo/GAP/) a method for discriminating amyloid forming peptides and amorphous peptides using a data set of 139 amyloids and 168 amorphous peptides. pasta (http://protein.bio.unipd.it/pasta2/about.html) predicts the most aggregation‐prone portions and the corresponding β‐strand intermolecular pairing for multiple input sequences. aggrescan (http://bioinf.uab.es/aggrescan/) is based on an aggregation‐propensity scale for natural amino acids derived from *in vivo* experiments and on the assumption that short and specific sequence stretches modulate protein aggregation.

a Taken from Ref. 19.

**Figure 1:**
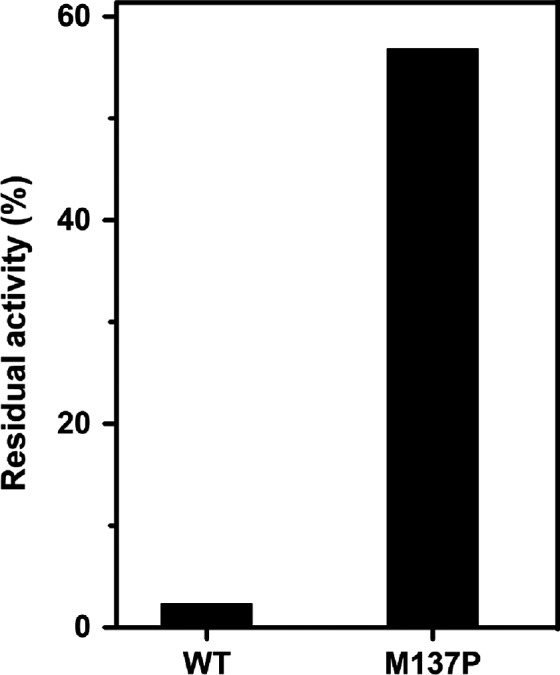
Reversibility of lipase variants. Residual activity, measured at room temperature following heat treatment at 65 °C for 20 min and cooling at 4 °C for 30 min.

The enzyme activity of 11 purified mutants used in this study is in the range of 0.5–1.8 fold that of the wild‐type protein 19. We generated aggregates from pure proteins of single mutants and wild‐type lipase by heating at 65 °C for 20 min. At room temperature the kinetics of aggregation of wild‐type and the mutant lipases are indistinguishable. Completeness of aggregation in lipase mutants was estimated by measuring residual activity at room temperature in the supernatants by separating the aggregates by centrifugation. Zero residual activity was seen in the supernatants of the wild‐type lipase in concurrence with the earlier reports. Except M137P, all the other mutants showed nearly zero residual activity like wild‐type lipase (Fig. 1). M137P showed ~ 57% residual activity after subjecting to 65 °C. As *T*_m_ of M137P (> 61 °C) was highest of all the mutants, it is possible that heating at 65 °C might not be high enough temperature to cause complete unfolding and aggregation or the mutation allows partial refolding into native form upon cooling. Hence, we also treated the mutant, M137P, at 70 and 80 °C and observed residual activity, ~ 38 and 31%, respectively, in the supernatants. Results from residual activity measurements suggested that none of these single mutations (except M137P) could resist unfolding mediated aggregation. In an earlier study, we have estimated the kinetics of inactivation for each of the mutants by incubating them at 55 °C and monitoring the activity at various time points till the activity was reduced to 20% of the initial activity. The inactivation kinetics of the eleven mutants is provided in Table 1 (19). The inactivation rates range from 3 min (wt) to 2600 (M137P) min, suggesting large differences in kinetic stabilities of the mutants.

**Figure 2:**
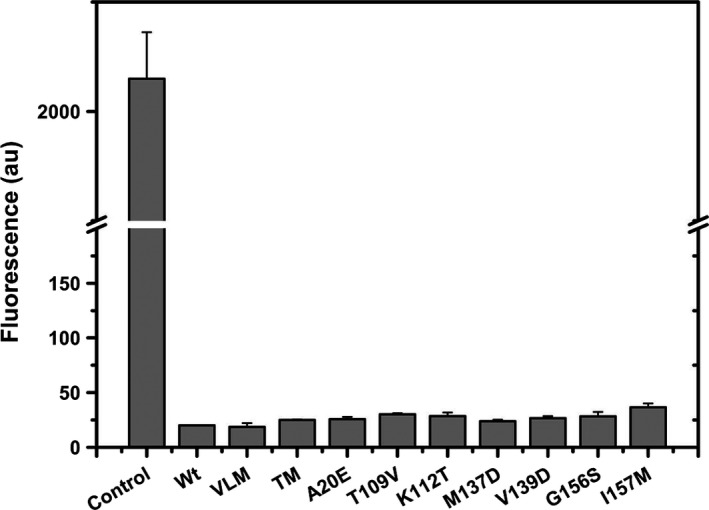
Binding of TfT with aggregates of various mutants. Aggregates of lipase and its mutants were incubated with 1 μm of TfT and fluorescence was taken. Equal amount of insulin (50 μg) was heat treated at low pH to produce amyloid aggregates and was used as a positive control.

We investigated whether the aggregates formed upon heating of lipase and its mutants are amyloid in nature by monitoring their binding with TfT, a dye that binds to β‐sheet rich structures such as amyloids 17. As a positive control we used amyloid fibrils generated by heating insulin at low pH, which is known to form amyloid aggregates. The data in Fig. 2 show that amyloids formed by insulin bind to TfT strongly, whereas the aggregates formed by the mutants or wild‐type lipase did not bind to TfT. This observation suggests that the aggregates are not fibrillar but probably amorphous in nature.

**Figure 3:**
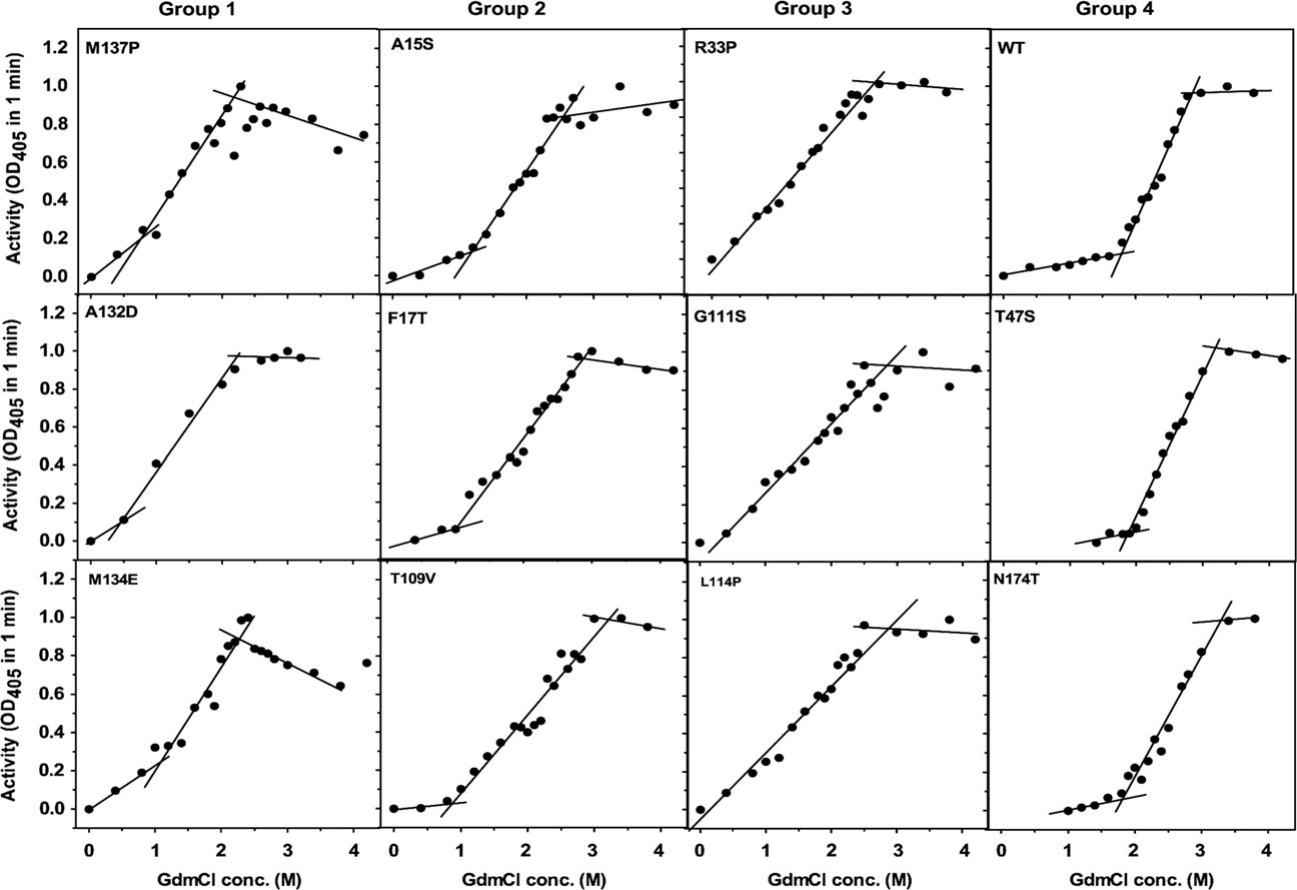
Stability of aggregates formed by lipase variants. Residual activity, measured at room temperature, after aggregates of lipase variants were dissolved in various concentration of GdmCl and diluted to < 1 m GdmCl.

**Figure 4:**
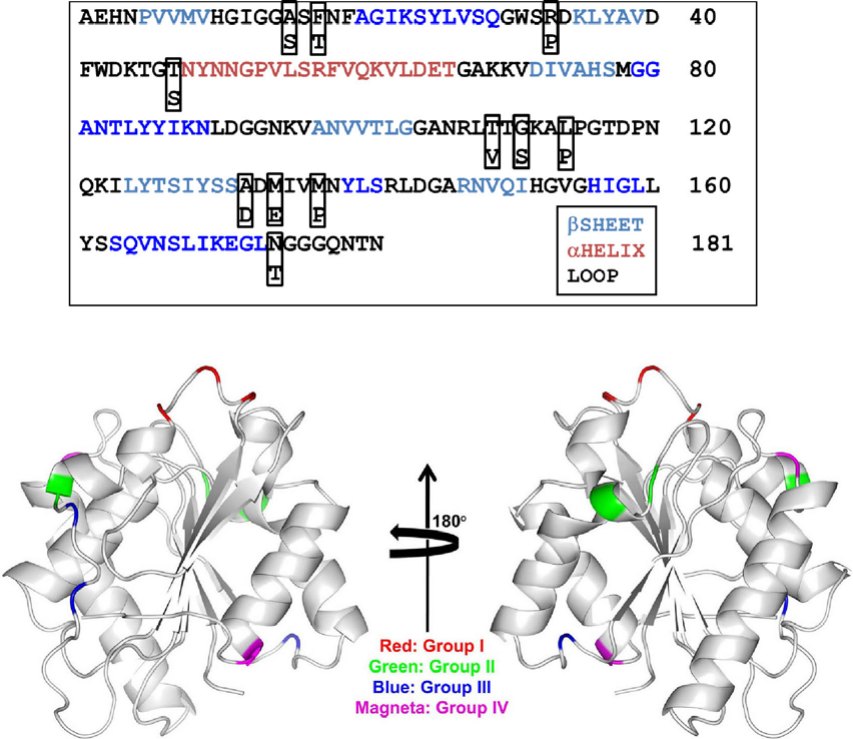
Location of mutations on lipase structure. (Top) The sequence of lipase along with secondary structure regions and location of mutations. (Bottom) Various mutations categorized based on their profiles of aggregate dissolution in GdmCl are shown in different colours. Catalytic residues (S77, D133 and H156) are also shown as stick model in elemental colour.

Protein aggregates could be disaggregated in the presence of denaturants such as GdmCl or urea. This process is often followed in recovering protein from inclusion bodies. To understand whether the aggregates formed by these 11 mutants differ in their response to GdmCl, we diluted the aggregates in various concentrations of GdmCl and by measuring the activity the effect of GdmCl on each mutant was monitored. GdmCl is a chaotrope, which is known to weaken the hydrophobic interaction and is a popular protein denaturant. We also established that GdmCl unfolded wild‐type lipase and it regains native structure and activity upon renaturation by diluting the denaturant (data not shown). Aggregates of all the mutants were completely dissolved at high GdmCl concentrations (> 3 m), however, the activity recovery profiles varied among mutants (Fig. 3). The heat induced aggregates of all the mutants and the wild‐type lipase are inactive in the absence of GdmCl. Aggregate of wild‐type lipase did not recover activity up to ~ 1.5 m GdmCl but completely recovered activity at ~ 3 m concentration. Recovery of activity was also visually observed by the absence of suspended aggregates. Aggregates of various mutants showed noticeable variations in response towards GdmCl treatment. Based on the profiles of activity recovery in the presence of GdmCl, we could categorize all the mutants and wild‐type profiles broadly into four groups (Fig. S1). The data on the activity along with the errors associated with the measurement are included as Fig. S2. Group I mutants namely A132D, M134E and M137P recovered activity at low concentration of GdmCl, even at 0.5 m GdmCl, and complete dissolution could be seen at ~ 2 m GdmCl. For aggregates of group II mutants (A15S, F17T and T109V), dissolution started at ~ 1 m GdmCl and was complete at ~ 2.5 m. For aggregates of group III mutants (R33P, G111S and L114P), although dissolution could be noticed even at 0.5 m GdmCl, completion could be seen only at ~ 3 m. Group IV consists of mutants (T47S and N174T), whose aggregate showed dissolution pattern similar to wild‐type lipase; hence the behaviour of these mutants is considered to be similar to that of wild‐type. Next, we mapped the positions of various single mutants on wild‐type crystal structure (Fig. 4). Although the grouping of mutants is based on similarity of profiles of the activity recovery, we found that mutants belonging to a group tend to occupy the same region on the protein. Mutations A132D, M134E and M137P, which belong to group I were located on the same loop (connecting β7 to αE) and occupy the same region on protein. Group II mutations A15S and F17T also shared the same region of lipase that is, on a loop that connects β3 strand to G1‐αA continuous helix. Likewise, group III mutations G111S and L114P were also on the same loop, a 14 residue long and connect proceeding G4, a 3_10_ helix to β7 strand. Only exceptions were mutations T109V and R33P, which although belong to group II and III respectively, were located away from the other mutations of the same group. Group IV mutations, which did not hamper the aggregation behaviour of lipase did not occupy the same region of protein like other group's mutants but were located at different regions of protein.

We have analysed the lipase sequences containing the 11 mutations to four popular softwares (tango, gap, pasta and aggrescan) that predict aggregation proneness in proteins. Each of these programs approaches the aggregation from different perspectives (See Table legend). The data obtained with these data bases are presented in Table 1. Apparently each of the softwares predicts the aggregation propensity of these mutant sequences differently; however, some consensus is seen with M137P and M134E substitutions. The consensus among softwares is high on combination of mutations (see below).

### Creation of nonaggregating mutant

**Figure 5:**
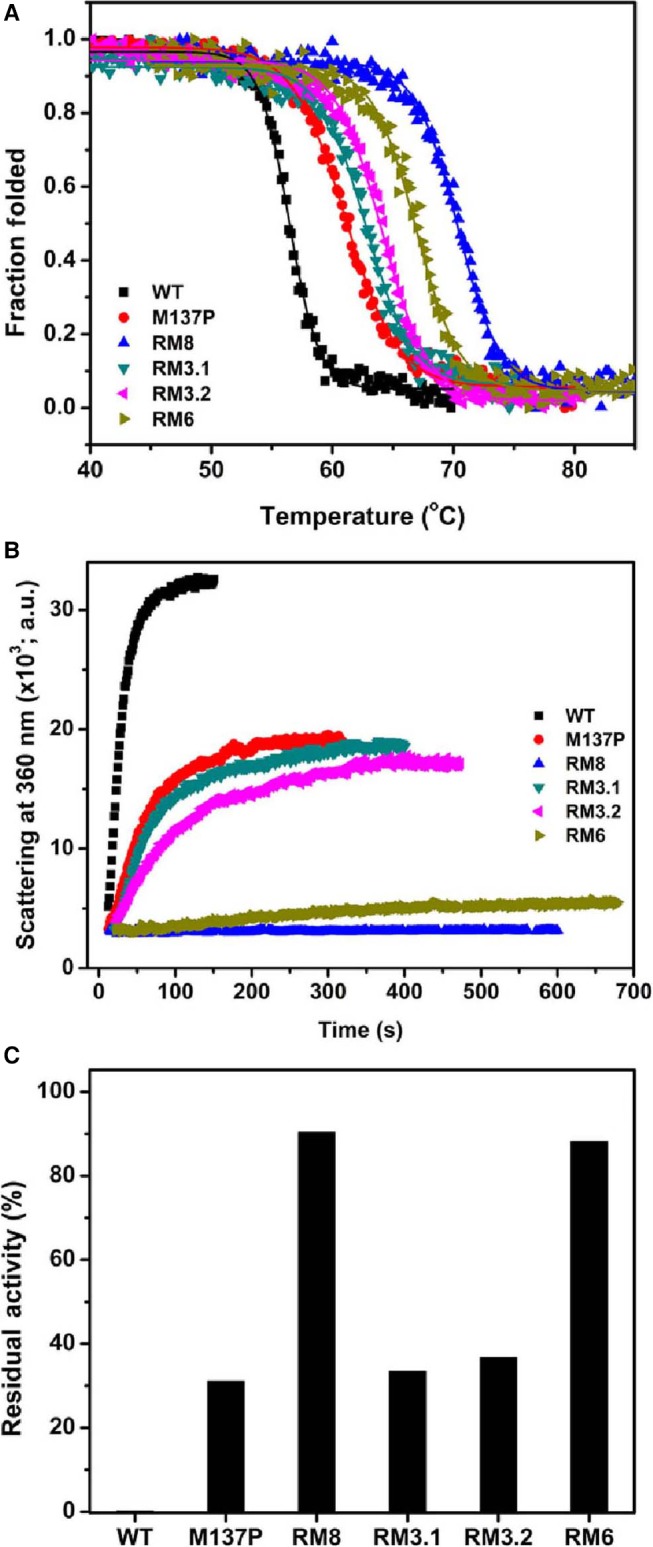
Stability and aggregation resistance of lipase mutants. (A) Thermal unfolding profile of various lipase mutants. Incorporation of mutations increases the stability (*T*_m_) of lipase variants. (B) Light scattering profile of various lipase variants at their respective *T*_m_ + 3 °C temperature. Wild‐type lipase scatters most while RM8 scatter least suggesting their corresponding heavy and least aggregation behaviour upon heat treatment. Other mutants showed intermediate scattering (hence, intermediate level of aggregation). (C) Residual activity, measured at room temperature following heat treatment at 80 °C for 20 min and cooling at 4 °C for 30 min. RM8 and RM6 maintains near complete activity suggesting prevention of protein aggregation while wild‐type lipase do not shows near zero residual activity suggesting its complete aggregation upon heat treatment.

We speculated that mutants belonging to a group may undergo the same unfolding process. Hence, we recombined all the mutations in these three loops (A15S, F17T, T109V, G111S, L114P, A132D, M134E and M137P), except mutations of group IV (T47S and N174T), into one gene using site‐directed mutagenesis, creating a recombinant lipase named RM8, harbouring a total of eight mutations. Thermal denaturation of RM8 was monitored using circular dichroism (CD) using a spectropolarimeter equipped with a Peltier controller. As can be seen in Fig. 5A, the melting temperature (*T*_m_) of RM8 was ~ 70.3 °C which is ~ 14.2 °C higher than that of wild‐type lipase (*T*_m_ ~ 56.1 °C). This mutant showed near complete prevention of thermal aggregation as monitored by light scattering experiment and residual activity measurement following heat treatments at 80 °C (Fig. 5B,C). Apparently, blocking the local unfolding of various loops by incorporating stabilizing mutations prevented the thermal aggregation of this mutant lipase, while simultaneously improving the protein stability significantly.

### Minimizing number of mutations

Next, we attempted to obtain a nonaggregating form of lipase by including minimum number of mutations. In the first effort, we tried to combine single mutations from each of the groups I, II and III. We have selected F17T, G111S and M137P. Selection of M137P mutation was based on its strong role in preventing aggregation compared to the other two mutations A132D and M134E in the group. Selection of F17T and G111S, from Group II and III respectively, was arbitrary. These three mutations were recombined using site‐directed mutagenesis to create a triple mutant named RM3.1. In the second instance, we have taken the simple and straightforward rationale of combining the mutations which were shown to affect the aggregation the most. Hence, we created another triple mutant named RM3.2, harbouring A132D, M134E and M137P mutations on the same loop. Both the triple mutants were assessed for their role in prevention of aggregation. Unfortunately, the two mutant lipases showed any significant improvement over M137P single mutations despite increasing protein stability (Fig. 5). We inferred that while in RM3.1 mutations F17T and G111S did not have strong enough stabilizing effect to block the local unfolding. Blocking the local unfolding of a single loop as in RM3.2 was also not effective to prevent aggregation.

In the next attempt to minimize the number of mutation, we recombined M137P with all the mutations from the other two loops (A15S, F17T, T109V, G111S and L114P) creating a mutant named RM6, harbouring a total of six mutations. As seen in Fig. 5, this mutant showed near complete resistance to thermal aggregation with concomitant increase in protein stability (~ 10.8 °C increase in *T*_m_ over wild‐type lipase). Evidently RM6 is similar to RM8 in thermal stability and in its ability to resist aggregation in tested conditions.

## Discussion

Aggregation in proteins has attracted considerable attention due to its importance in protein biotechnology and also in health. The interactions that lead to aggregation in proteins are similar to natural processes, such as hydrophobic, ionic interactions etc. that are involved in folding of proteins after synthesis. One of the strategies to decrease aggregation tendencies in proteins is to increase the stability of the protein. The underlying assumption to this approach is that at equilibrium, native proteins coexist with unfolded state, and by shifting the bias towards the folded state, by increasing the free energy of unfolding, proteins could be retained in the native state 20. Protein dynamics in the native state play a critical role in native to unfolded state transition. By dampening the protein dynamics by stabilizing the protein, for example, by increasing the number or quality of intramolecular bonds, the aggregation tendency could be reduced. The most dynamic parts of proteins are loops or unordered regions of the protein that occupy the surface of the protein. Loops, due to their flexibility, facilitate the important conformational changes necessary in protein function. On the same count, loops are also susceptible to changes in the ambient conditions such as temperature, pH etc. Many protein engineering studies have demonstrated that by stabilizing the loops, both kinetic and thermodynamic stability of a protein could be improved 7. In this study, we have employed 11 single amino acid mutants of lipase which differ in their melting temperatures and free energy of denaturation (Table 1). These mutants differ from wild‐type by 1–6 °C in *T*_m_ and by < 1Kcal·mol^−1^ in ΔG_unfold_ 19. Kinetic stability, as monitored by loss of activity at 55 °C, however, was vastly different between mutants (Table 1). While *t*_1/2_ of wild‐type is 3 min, the half‐ life of M137P is 2617 min.

As these mutations are located on various regions of the lipase and differ in kinetic stability, we presumed the unfolding rates would lead to formation of aggregates that are dissimilar to each other. To evaluate this, we have subjected these aggregates, formed by heating the pure proteins to 60 °C, to various concentrations of denaturant, GdmCl. The resulting activity profiles are different for each of the mutants. The differences in activity profiles may originate due to: (a) Dissimilar unfolding kinetics among the mutants involving different unfolding intermediates; (b) Possible differences in disaggregation of aggregates in the presence of GdmCl; (c) Unfolding and refolding abilities, with respect to activity, of the mutants in the presence of GdmCl. All three processes would have played a role in the observed activity profiles of aggregates in GdmCl. From our earlier studies, the differences in unfolding/refolding abilities of the mutants are distributed in a narrow range of denaturant and thus these differences may play a lesser role in the observed activity profiles. A clear difference between mutants is their kinetic stability, which ranges from 6 min (A132D) to 2617 min (M137P). These differences could manifest in dissimilar unfolding pathways leading to different aggregate forms. It is interesting to notice that Group I mutants show very long half‐life (more than 190 min), except for A132D, Group II mutants intermediate half‐ life in the range of 20–40 min and Group III mutants show the least half‐life (< 10 min). As there are some exceptions (A132D), the observed similarity in half‐lives could at best be suggestive of the role of these regions in thermal unfolding. Characterizing aggregates by size was not possible in our case as the sizes of all aggregates are very large (by DLS). We have tested the binding of TfT, a well‐known indicator of amyloids. None of the aggregates bind to TfT to any appreciable extent, indicating the nonamyloid nature of these aggregates.

The grouping of the activity profiles (Fig. 3) is based on similarities, primarily based on GdmCl scale that is, taking into consideration the beginning of activity and the length of transition. The adjacent location of the mutants belonging to each group suggests that the location may have a role in unfolding process and consequent aggregation. Combining the mutations in four possible ways that is, all mutations (*n* = 8) or one from each group (*n* = 3) or mutations in one loop (*n* = 3) or best mutation M137P with other five mutations, we observed RM6 is the best mutant with least aggregation and with the least number of mutations.

Excellent studies on amino acid substitutions and aggregation kinetics have tremendously improved our understanding of the physical basis of aggregation in proteins 1, 2, 3. The derived assignment of aggregation tendencies to amino acids or short sequences led to a number of useful softwares that predict aggregation tendencies in proteins. Most of these data were derived from room temperature aggregation kinetics obtained from different proteins. In our case the aggregation is thermally induced and to that extent the scores obtained by using these softwares may not be appropriate.

Based on activity recovery profiles obtained on thermally aggregated mutants of lipase, it is apparent that the aggregates formed by different mutants are different. This differential susceptibility of aggregates to GdmCl may have originated from the differences in kinetic stability of the mutants. This study supports suggestions that unfolding may initiate at different locations on a protein and each of these unfolding hotspots leads to formation of intermediates that eventually form aggregates that are different. As mutations located close to each other apparently result in similar inactivation kinetics and activity profiles of aggregates in GdmCl, we suggest that unfolding in lipase may initiate at three different locations. In addition, combining the mutations from each of these regions has resulted in a mutant that is very stable and shows excellent aggregation resistance.

## Conclusion

Protein aggregation is a wasteful process in protein suspensions and formulations 21. Although considerable empirical strategies have been developed to overcome this process, understanding of protein aggregation is still incomplete. Aggregation of proteins is considered to be initiated by nonspecific intermolecular interactions that originate from normal protein dynamics. By dampening the protein fluctuations the process could be partly mitigated. By presuming that alteration of formation of these intermediates could lead to aggregates with altered properties, we have probed the aggregates, formed from 11 single mutants of a lipase, by ‘denaturing’ them in GdmCl. The denaturation profiles of these 11 mutants could be assigned to four groups. Out of four groups mutations in three groups are also near neighbours on the protein. Inclusion of mutants from each group into a single protein resulted in very stable protein that is also resistant to aggregation. We believe our method of capturing effect of mutations on protein aggregation and identifying mutations altering local unfolding events can be applied to create nonaggregating and stable versions of proteins.

## Author contributions

ZK has supervised the work and analysed the data, VK has purified enzymes and performed initial characterization, KKD has performed the experiments, KS has generated the mutants and NMR has written and conceptualized the work.

## Supporting information


**Fig. S1.** Tentative grouping of the activity recovery profiles of the 11 mutants of lipase.
**Fig. S2.** Data presented as Fig. 3 in the main text are presented along with the errors associated with each data point.

## References

[1] Chiti F and Dobson CM (2006) Protein misfolding, functional amyloid, and human disease. Annu Rev Biochem 75, 333–366. 16756495 10.1146/annurev.biochem.75.101304.123901

[2] Chiti F and Dobson C (2009) Amyloid formation by globular proteins under native conditions. Nat Chem Biol 5, 15–22. 19088715 10.1038/nchembio.131

[3] Castillo V , Graña‐Montes R , Sabate R and Ventura S (2011) Prediction of the aggregation propensity of proteins from the primary sequence: aggregation properties of proteomes. Biotechnol J 6, 674–685. 21538897 10.1002/biot.201000331

[4] Bondos SE and Bicknell A (2003) Detection and prevention of protein aggregation before, during, and after purification. Anal Biochem 316, 223–231. 12711344 10.1016/s0003-2697(03)00059-9

[5] Wang W (2005) Protein aggregation and its inhibition in biopharmaceutics. Int J Phar 289, 1–30. 10.1016/j.ijpharm.2004.11.014 15652195

[6] Wang W , Nema S and Teagarden D (2001) Protein aggregation–pathways and influencing factors. Int J Pharm 390, 89–99. 10.1016/j.ijpharm.2010.02.025 20188160

[7] Matthews CR (1993) Pathways of protein folding. Ann Rev Biochem 62, 663–683. 10.1146/annurev.bi.62.070193.003253 8352599

[8] Horwich A (2002) Protein aggregation in disease: a role for folding intermediates forming specific multimeric interactions. J Clin Invest 110, 1221–1232. 12417558 10.1172/JCI16781 PMC151620

[9] Murphy RM and Roberts CJ (2013) Protein misfolding and aggregation research: some thoughts on improving. Biotechnol Prog 29, 1109–1116. 24124114 10.1002/btpr.1812

[10] Rajan RS , Illing ME , Bence NF and Kopito RR (2001) Specificity in intracellular protein aggregation and inclusion body formation. PNAS 98, 13060–13065. 11687604 10.1073/pnas.181479798 PMC60824

[11] Acharya P , Rajakumara E , Sankaranarayanan R and Rao NM (2004) Structural basis of selection and thermostability of laboratory evolved *Bacillus subtilis* lipase. J Mol Biol 341, 1271–1281. 15321721 10.1016/j.jmb.2004.06.059

[12] Ahmad S , Kamal MZ , Sankaranarayanan R and Rao NM (2008) Thermostable *Bacillus subtilis* lipases: in vitro evolution and structural insight. J Mol Biol 381, 324–340. 18599073 10.1016/j.jmb.2008.05.063

[13] Kamal MZ , Ahmad S , Molugu TR , Vijayalakshmi A , Deshmukh MV , Sankaranarayanan R and Rao NM (2011) In vitro evolved non‐aggregating and thermostable lipase: structural and thermodynamic investigation. J Mol Biol 413, 726–741. 21925508 10.1016/j.jmb.2011.09.002

[14] Markwell MA , Haas SM , Tolbert NE and Bieber LL (1981) Protein determination in membrane and lipoprotein samples: manual and automated procedures. Methods Enzymol 72, 296–303. 6796803 10.1016/s0076-6879(81)72018-4

[15] Ban T , Hamada D , Hasegawa K , Naiki H and Goto Y (2003) Direct observation of amyloid fibril growth monitored by thioflavin T fluorescence*210. J Biol Chem 278, 16462–16465. 12646572 10.1074/jbc.C300049200

[16] Benjwal S , Verma S , Rohm KH and Gursky O (2006) Monitoring protein aggregation during thermal unfolding in circular dichroism experiments. Protein Sci 15, 635–639. 16452626 10.1110/ps.051917406 PMC2249783

[17] Duy C and Fitter J (2005) Thermostability of irreversible unfolding alpha‐amylases analyzed by unfolding kinetics. J Biol Chem 280, 37360–37365. 16150692 10.1074/jbc.M507530200

[18] Khurana R , Gillespie JR , Talapatra A , Minert LJ , Ionescu‐Zanetti C , Millett I and Fink AL (2001) Partially folded intermediates as critical precursors of light chain amyloid fibrils and amorphous aggregates. Biochemistry 40, 3525–3535. 11297418 10.1021/bi001782b

[19] Ahmad S , Kumar V , Ramanand KB and Rao NM (2012) Probing protein stability and proteolytic resistance by loop scanning: a comprehensive mutational analysis. Protein Sci 21, 433–446. 22246996 10.1002/pro.2029 PMC3375443

[20] Costanzo JA , O'Brien CJ , Tiller K , Tamargo E , Robinson AS , Roberts CJ and Fernandez EJ (2014) Conformational stability as a design target to control protein aggregation. Protein Eng Des Sel 27, 157–167. 24722670 10.1093/protein/gzu008

[21] Fink AL (1998) Protein aggregation: folding aggregates, inclusion bodies and amyloid. Fold Des 3, R9–R23. 9502314 10.1016/S1359-0278(98)00002-9

