# Supplementary material for: Mutational probing of protein aggregates to design aggregation‐resistant proteins
Source: FEBS Open Bio. 2016 Jan 4;6(2):126–34. doi: 10.1002/2211-5463.12003 (PMC4821347; doi:10.1002/2211-5463.12003)

**Supplementary Material for the manuscript by Zahid et al**

Supplementary Fig. 1: Tentative grouping of the activity recovery profiles of the 11 mutants of lipase

Supplementary Fig. 2: Categorization of lipase mutants based on their effect on aggregation.


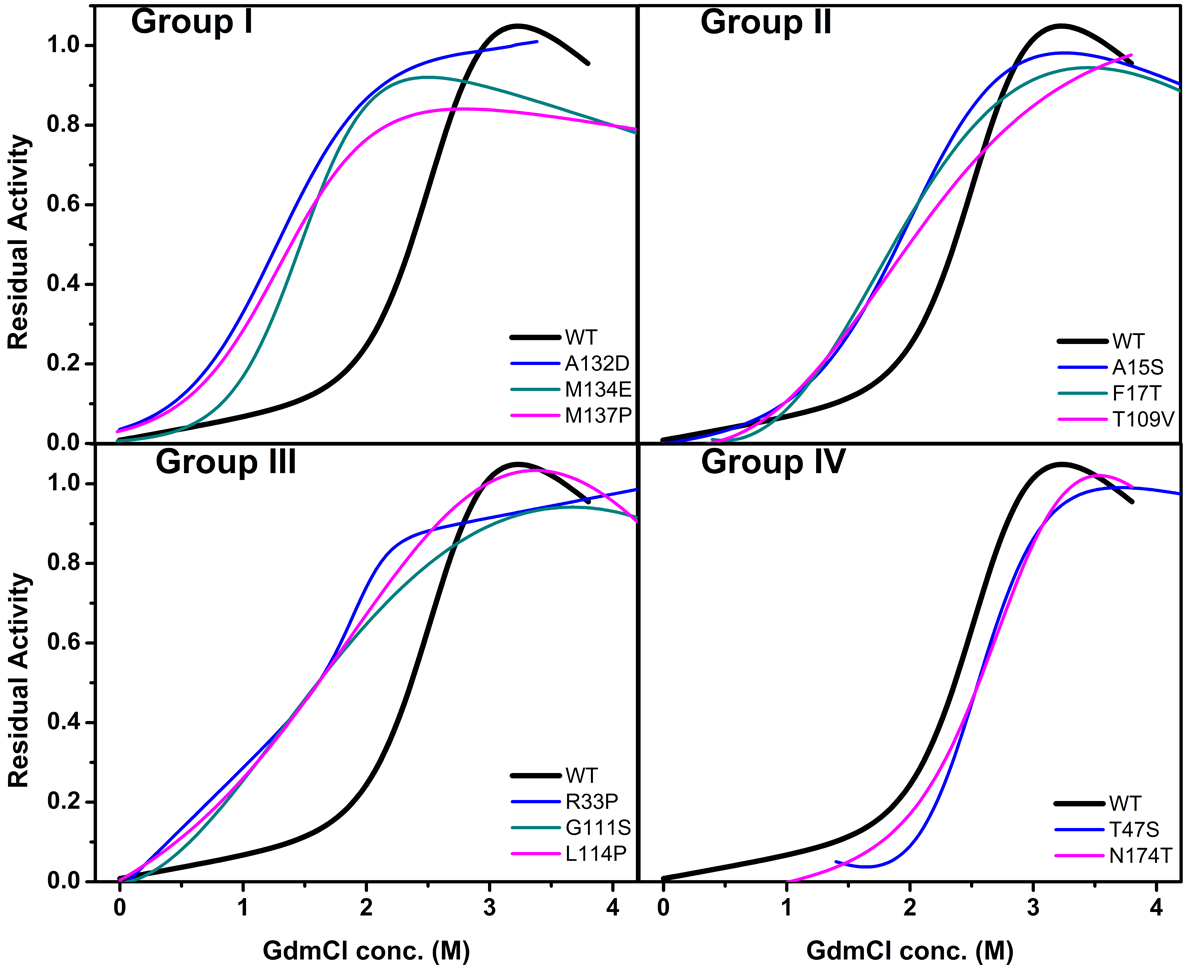


Profiles of dissolution of aggregates of various lipase variants by GdmCl were grouped based on their similarities.

Supplementary Fig. 2: Data presented as Fig.3 in the main text is presented along with the errors associate with each data point


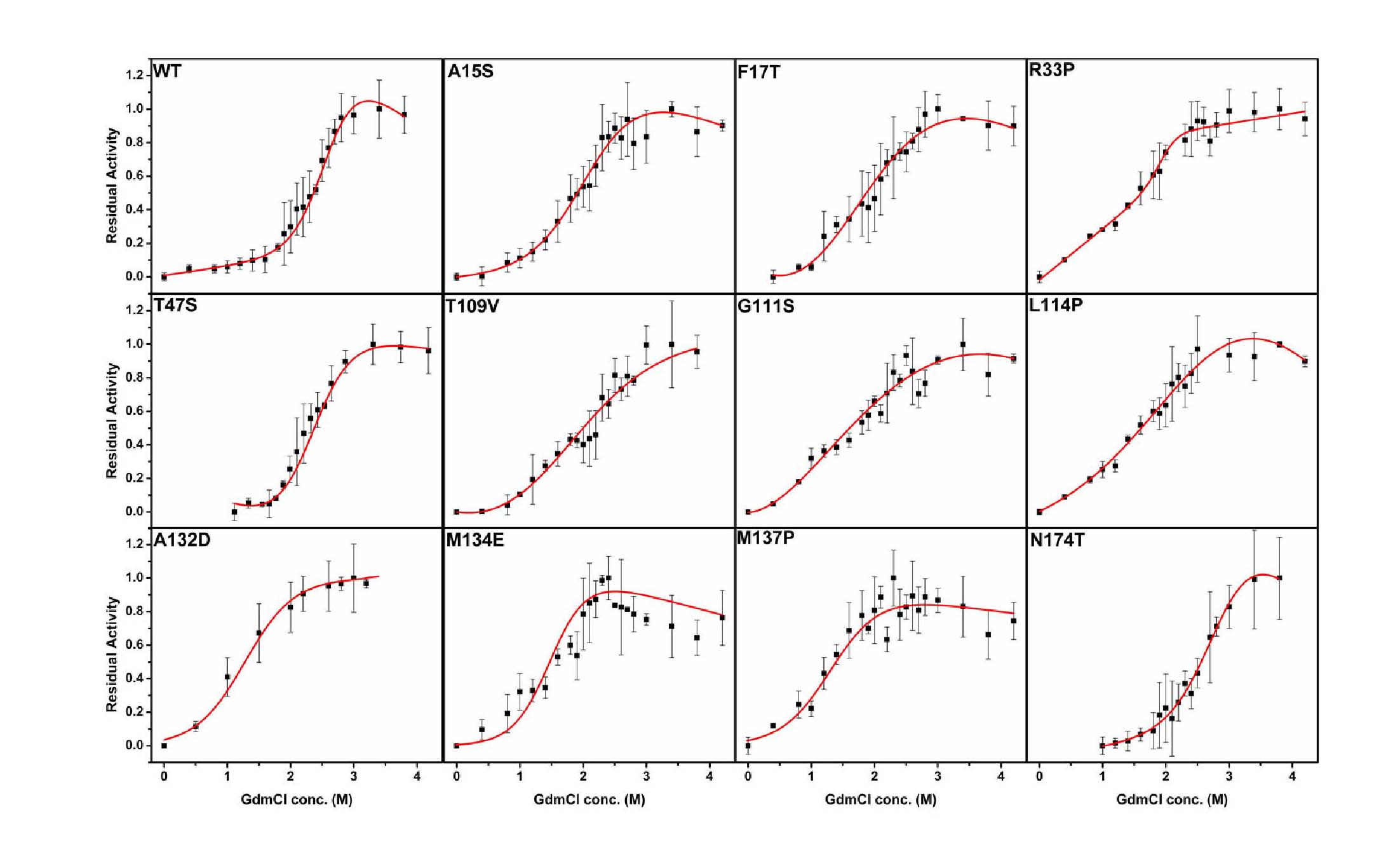

Supplement: Supplementary file 1 — Fig. S1. Tentative grouping of the activity recovery profiles of the 11 mutants of lipase. Fig. S2. Data presented as Fig. 3 in the main text are presented along with the errors associated with each data point. [file FEB4-6-126-s001.doc]
